# Endoscopic and Pathological Examinations of Early-Signet-Ring Carcinoma in the Stomach

**DOI:** 10.3390/healthcare13212689

**Published:** 2025-10-23

**Authors:** Zhao Liang, Liang Zheng, Jia Cao

**Affiliations:** 1 Digestive Endoscopy Center, East Hospital, Tongji University School of Medicine, Shanghai 200090, China; lz2114490@outlook.com; 2 School of Medicine, Tongji University, Shanghai 200092, China; zhengliang@tongji.edu.cn

**Keywords:** early gastric cancer, signet-ring cell carcinoma of the stomach, risk factors, endoscopic diagnosis

## Abstract

**Objective:** Early-signet-ring cell carcinoma has a low malignancy and good prognosis, while advanced signet-ring cell carcinoma has high malignancy and high mortality. So, we need to understand the risk factors of early-signet-ring cell carcinoma, analyze the relationship between early gastric signet-ring cell carcinoma and non-Signet-ring cell carcinoma, and between pure signet-ring cell carcinoma and mixed signet-ring cell carcinoma, in order to provide the basis for the early diagnosis and treatment of signet-ring cell carcinoma. **Methods:** In this study, a retrospective analysis of 424 cases of early gastric cancer that underwent endoscopic submucosal dissection and surgical treatment between March 2019 and March 2023 in Shanghai Oriental Hospital was carried out. Among the cases, the two groups, namely, the signet-ring cell carcinoma and non-signet-ring cell carcinoma group, and the pure signet-ring cell carcinoma and mixed signet-ring cell carcinoma group, were compared and analyzed. With the help of logistic regression analysis, gender, age, smoking history, alcohol consumption history, tumor site, pathological characteristics, disease progression, tumor size, infiltration depth, and H. pylori infection were investigated between the two groups. **Result:** The results of the univariate regression analyses in the signet-ring cell carcinoma and non-signet-ring cell carcinoma groups showed that being female (*p* = 0.001), age < 60 years (*p* = 0.001), with cancer foci in the middle part of the stomach (*p* = 0.001), and with a mixed type of cancer foci (*p* = 0.007) were the risk factors for signet-ring cell carcinoma. In the multifactorial regression analysis, age < 60 years (OR = 1.037, CL = 1.008–1.067, *p* = 0.012), cancer foci in the middle part of the stomach (OR = 2.094, CL = 1.488–2.948, *p* = 0.001), mixed-type patients (OR = 0.702, CL = 0.519–0.951, *p* = 0.022), and women (OR = 0.421, CL = 0.254–0.698, *p* = 0.001) were the risk factors for signet-ring cell cancer. These are independent risk factors for signet-ring cell carcinoids. Univariate regression analysis on the pure and mixed signet-ring cell carcinoma groups showed that Helicobacter pylori infection (*p* = 0.001), Kimura–Takemoto classification O1–O3 (*p* = 0.013), flat and concave types (*p* = 0.004), and age < 60 years (*p* = 0.013) were risk factors affecting the development of pure signet-ring cell carcinoma. In the multifactorial regression analysis, age (OR = 0.233, CL = 0.059–0.930, *p* = 0.039) was the main independent risk factor for pure signet-ring cell carcinoma. **Conclusions:** Age < 60 years, cancer foci located in the middle of the stomach, mixed type, and female are associated with the development of early gastric signet-ring cell carcinoma; age < 60 years is related to the development of pure signet-ring cell carcinoma, so we need to pay attention to these clinical and pathological factors to prevent the growth of ring cell carcinoma.

## 1. Introduction

Gastric cancer is a prevalent malignant tumor in our daily lives [1]. Gastric cancer ranks fifth in incidence among all malignancies, and the mortality rate is the third highest [2]. Gastric cancer has seriously affected people’s lives and health. The World Health Organization classifies gastric cancer in detail to include papillary carcinoma, tubular carcinoma, mucinous carcinoma, undifferentiated carcinoma, and signet-ring cell carcinoma [3]. Gastric signet-ring cell carcinoma (SRCC) is also a special type of gastric cancer containing mucus, accounting for 3.4% to 39% of primary gastric cancers [4]. Gastric signet-ring cell carcinoma derives its name from its distinctive cells, which contain abundant mucus, pushing the nucleus to one side [5], resembling a signet ring. According to the proportion of mucus components, gastric cancer can be classified into signet-ring cell carcinoma (SRCC) and non-signet-ring cell carcinoma (NSRCC), and signet-ring cell carcinoma can be further divided into pure signet-ring cell carcinoma (PSRCC) and mixed signet-ring cell carcinoma (MSRCC) [6]. According to the classification of the World Health Organization, pure signet-ring cell carcinoma of the stomach is a very specific subtype of gastric cancer, characterized by a tumor composed entirely of signet-ring cells (more than 50%). Mixed signet-ring cell carcinoma of the stomach is a relatively rare subtype of gastric cancer, which is characterized by tumor tissue containing both signet-ring cell (usually 10–50%) [7] and non-signet-ring cell components (e.g., adenocarcinomas, papillary carcinomas, or undifferentiated carcinomas), and is associated with worse biological behavior and clinical outcomes compared to pure signet-ring cell carcinoma [8].

We mainly studied early gastric cancer patients who underwent ESD and surgery at Shanghai Dongfang Hospital from March 2019 to March 2023, to collect the basic clinical information of the patients with early gastric SRCCs; analyze the relationship between gastric SRCC and gastric NSRCC, and the relationship between gastric PSRCC and gastric MSRCC; to explore the factors affecting the occurrence of early gastric SRCC; and to provide a reference for the early diagnosis and treatment of early gastric SRCC.

## 2. Data and Methods

### 2.1. Object

This is a single-center retrospective study, and 424 patients with early gastric cancer who attended Shanghai Oriental Hospital between March 2019 and March 2023 were selected as subjects. All patients met the criteria for early gastric cancer as defined by the Japanese Gastric Cancer Association (JGCA), i.e., tumors confined to the mucosal or submucosal layer of the gastric wall, irrespective of their lymph node metastatic status, and the clinical data of these patients were exhaustively collected and analyzed.

This study’s inclusion criteria are as follows: (1) The postoperative pathological examination after ESD and surgical operation showed that the cancer lesion was limited to the mucosal layer and submucosal layer; (2) Imaging examination results show no distant metastasis; (3) Complete clinical data of the cases were obtained; and (4) No radiotherapy or chemotherapy was performed. The exclusion criteria for this study were (1) A history of malignant tumors in the digestive system; (2) Postoperative pathological examination showed that it was not early gastric cancer; (3) Presence of residual gastric cancer; and (4) Existence of surgical contraindications, making surgery impossible. Ethical clearance for the planning and execution of this study was obtained from the Medical Ethics Committee of the hospital, which granted exempted informed consent as this study was retrospective in nature.

### 2.2. Data Collection

The indicators included gender, age, lifestyle (smoking, drinking), family history, and tumor characteristics (size, location, shape, and depth of invasion). The Kimura–Takemoto classification was performed and H. pylori infection was examined, and background mucosal atrophy, etherization, inflammation, and immunohistochemical pathological details were evaluated. All pathological postoperative specimens were fixed by formalin immersion, paraffin embedding, and hematoxylin–eosin staining (HE).

### 2.3. Methods of Gastroscopy

Dimethylsiloxane powder (5.0 g) was administered before gastroscopy to eliminate foam in the stomach and allow a clearer field of view, and dicronin hydrochloride gel (20 mL) for local anesthesia of the pharynx was administered to reduce discomfort during the examination. A high-definition electronic gastroscope (GIF-HQ290, GIF-HQ290Z, GIF-H260 Olympus, Tokyo, Japan) was used for a biopsy and pathological examination if necessary. All gastroscopies were performed by professional endoscopists who had more than 10 years of clinical experience and had examined more than 3000 cases per year. When divergent cases existed, repeat biopsies, magnified endoscopic fine-tuning, or objective data support with the aid of AI-assisted diagnostic tools (e.g., lesion benignity and malignancy prediction models) were performed.

### 2.4. Criteria for Judging Observation Indicators

Age division: In this study, there were 424 cases of early gastric cancer. Of these, 292 were older than 60 years and 132 were younger than 60 years, with an average age of 64.7 years. Combined with World Health Organisation guidelines, the patients were categorized into two age-based groups: those aged 60 years and over, and those under 60 years.

Smoking history: Refers to individuals smoking more than one cigarette a day and for more than one consecutive year, divided into two groups of smoking history and no smoking history.

Drinking history: Drinking history is defined as drinking alcohol at least once a week for a period of time in the past (usually a year or more) and is categorized as present or absent.

Tumor size: In this study, 185 patients had tumors larger than 2 cm and 239 patients had tumors smaller than 2 cm, with a mean tumor size of 2.18 cm, combined with other gastric SRCC tumor size classifications in the relevant literature [9]. Therefore, patients were divided into two groups according to tumor size: one group with a tumor size larger than or equal to 2 cm and one group smaller than 2 cm.

Family history: A medical history of the patient admitted to the hospital. First-degree relatives (parents, brothers, and sisters) with gastric cancer history were divided into two groups: with and without gastric cancer.

H. pylori infection: H. pylori infection was classified into three categories: no infection, a past infection, and a current infection, with a urea breath test to detect the presence or absence of infection, and a serological test to determine the presence or absence of a past infection.

The classification of atrophic gastritis employed the Kimura–Takemoto system, distinguishing between two primary types: the tight (or close) type (C) and the open type (O). These types were further subdivided into grades for detailed categorization [10].

Closed type (C): The borderline between the anterior and posterior walls is closed on the side of the lesser curvature of the gastric body. It is divided into the following 3 grades:

C-1: The atrophic border is confined to the far side of the gastric angle incision and does not exceed the gastric angle.

C-2: The atrophic border exceeds the gastric angle but does not reach the midpoint of the side of the lesser curvature of the gastric body.

C-3: The atrophic border reaches or exceeds the midpoint of the lesser curvature of the gastric body, but does not reach the cardia.

Open type (O): The borderline between the anterior and posterior walls is not closed on the side of the lesser curvature of the gastric body. It is divided into the following 3 grades:

O-1: The atrophic border exceeds the gastric angle, is not closed on the side of the lesser curvature of the gastric body, and does not exceed the midpoint of the side of the greater curvature of the gastric body.

O-2: The atrophic border exceeds the midpoint of the greater curvature of the gastric body, but does not reach the cardia.

O-3: The atrophic border reaches the cardia or exceeds the cardia to involve the lower esophagus.

### 2.5. Statistical Methods

SPSS 22.0 was used as a statistical tool, χ2 was used to evaluate the results of the univariate analysis, and the variables with significant differences were identified as significant when *p* < 0.05 was less than *p* < 0.05 by logistic regression analysis.

Background: Mucosa atrophy, esterification grade, inflammation degree, mild to moderate activity grade, and trend chi-squared test were used to analyze whether there was statistical significance.

A risk prediction model was established, and the receiver operating characteristic curve (ROC) assessed the risk model’s predictive accuracy and discrimination.

**Figure 1:**
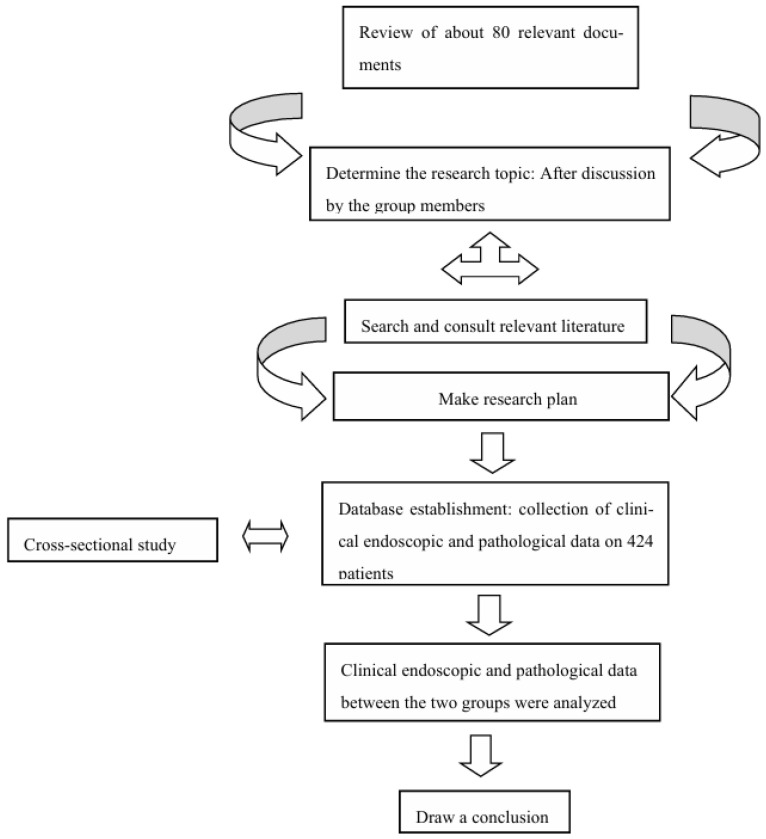
Sample-selected specific flow chart.

Figure 1 shows the screening process of 424 cases from this study.

## 3. Results

### 3.1. Basic Information of Patients

Among the 424 patients with early gastric cancer, 93 (21.9%) had a signet-ring cell carcinoma and 331 (78.1%) had a non-signet-ring cell carcinoma. Among them, 29 cases (31.2%) had a pure signet-ring cell carcinoma and 64 cases (68.8%) had a mixed signet-ring cell carcinoma. There were 281 males (66%) and 143 females (34%), with a male-to-female ratio of 1.97:1. The median age was 60 years (36–84 years).

### 3.2. Univariate and Multifactorial Results Obtained for Both Gastric SRCC and Gastric NSRCC

The results of the univariate regression analyses in the group of gastric SRCC and gastric NSRCC showed that being female (*p* = 0.001), age < 60 years (*p* = 0.001), with cancer foci in the middle part of the stomach (*p* = 0.001), and with a mixed type of cancer foci (*p* = 0.007) were the risk factors for signet-ring cell carcinoma. In the multifactorial regression analysis, age < 60 years (OR = 1.037, CL = 1.008–1.067, *p* = 0.012), cancer foci in the middle part of the stomach (OR = 2.094, CL = 1.488–2.948, *p* = 0.001), mixed-type patients (OR = 0.702, CL = 0.519–0.951, *p* = 0.022), and women (OR = 0.421, CL = 0.254–0.698, *p* = 0.001) were the risk factors for gastric SRCC. These are independent risk factors for gastric SRCC.

### 3.3. Univariate and Multivariate Analysis Results Obtained for Gastric PSRCC and Gastric MSRCC

Univariate regression analysis in the pure and mixed signet-cell carcinoma groups showed that Helicobacter pylori infection (*p* = 0.001), Kimura–Takemoto classification O1–O3 (*p* = 0.013), flat and concave types (*p* = 0.004), and age < 60 years (*p* = 0.013) were risk factors affecting the development of gastric PSRCC. On the multifactorial regression analysis, age (OR = 0.233, CL = 0.059–0.930, *p* = 0.039) was the main independent risk factor for gastric PSRCC.

**Figure 2:**
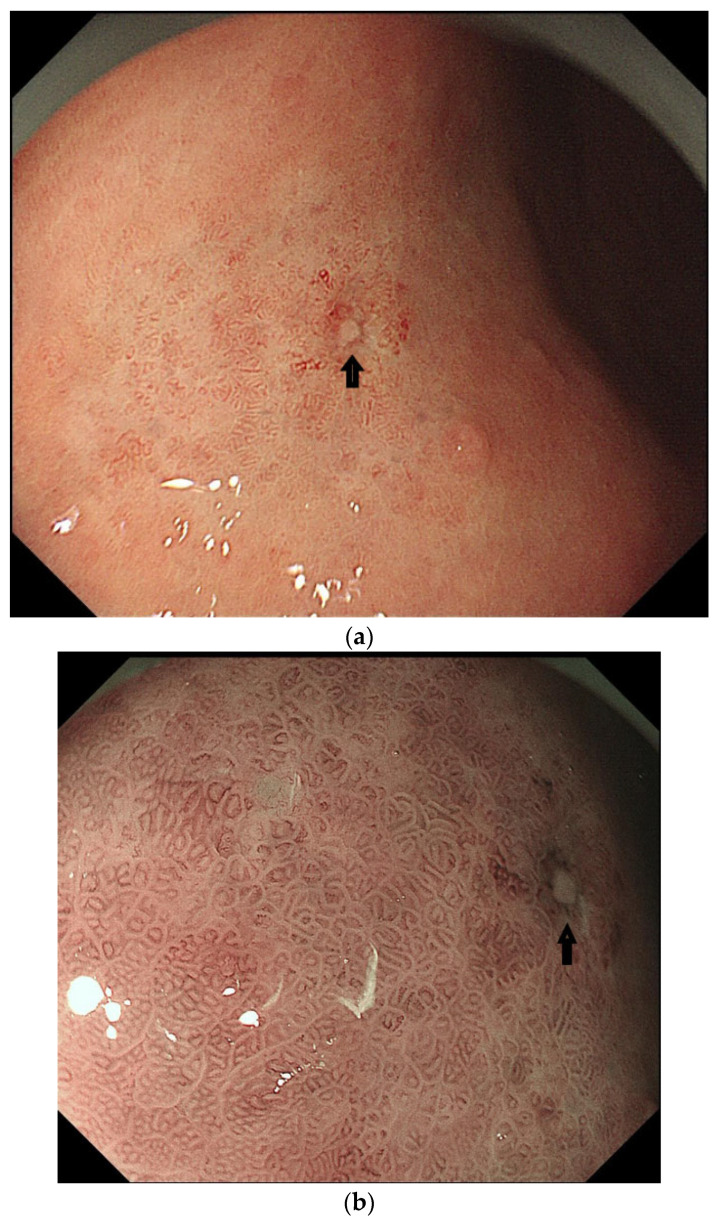

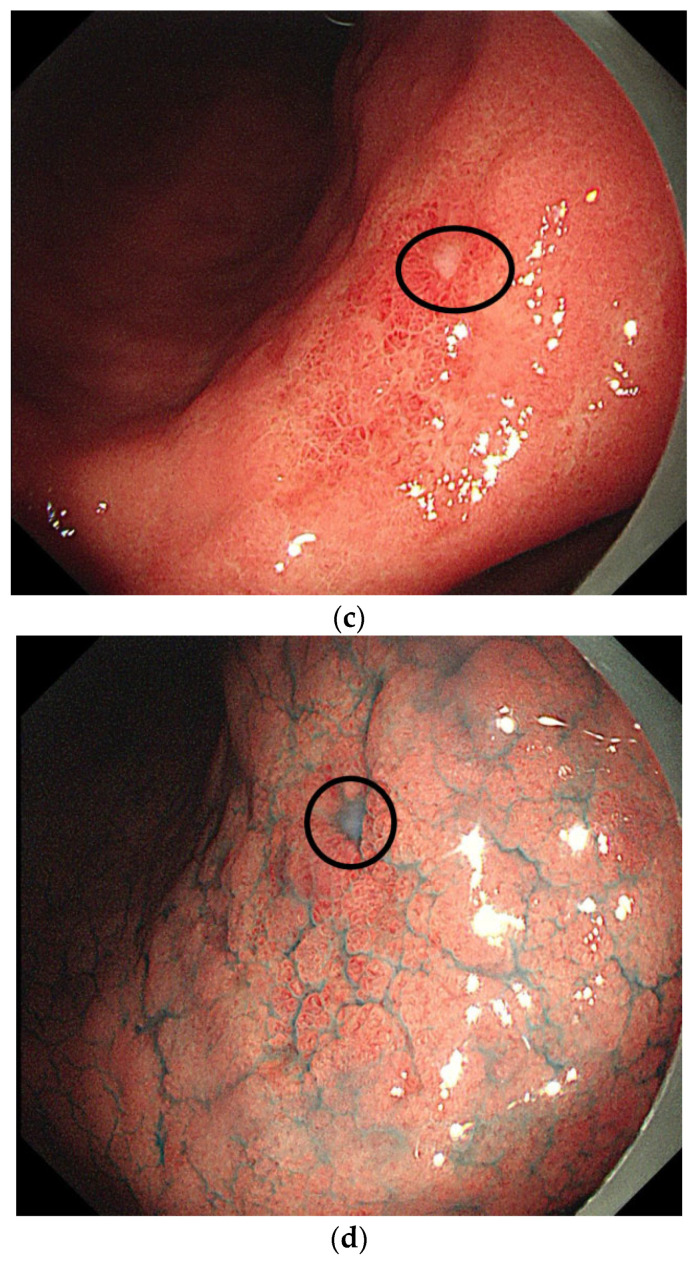
Endoscopic findings of early-signet-ring cell carcinoma in different parts of the stomach. (**a**) Gastric SRCC in white-light endoscopic picture. (**b**) Gastric SRCC in magnified + NBI endoscopic picture. (**c**) Gastric SRCC in white-light endoscopic picture. (**d**) Gastric SRCC in magnified + NBI endoscopic picture. The black circles and arrows are indicative of ring cell carcinoma.

**Figure 3:**
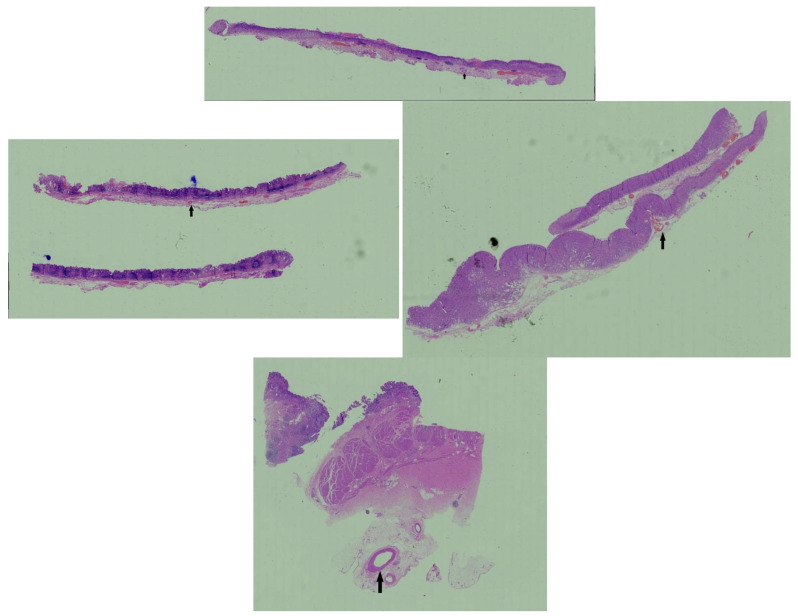
Pathological images of early gastric SRCC. The black arrow shows the position of the gastric SRCC.

Table 1 shows the comparison between the early gastric SRCC group and the gastric NSRCC group in the clinical endoscopy (Figure 2) and pathology (Figure 3), and the results show that there is statistical significance for gender, age, tumor site, and morphological manifestation distribution between the early gastric SRCC group and gastric NSRCC group (*p* < 0.05). Gastric SRCC in the middle third of the stomach was higher than that of gastric NSRCC (75%:34%, *p* = 0.001 < 0.05), and the proportion of a mixed-type differentiation of gastric SRCC was higher than that of gastric NSRCC (34%:20%, *p* = 0.007 < 0.05), with statistical significance.

Figure 2 shows the endoscopic images of signet-ring cell carcinomas. a: Mucosal erosion about 1.5 × 1.3 cm in size can be seen on the posterior wall of the gastric body near the gastric angle, with a red surface, and a biopsy scar can be seen (black arrow site). b: The NBI magnification shows the clear boundary of the lesion, an abnormal surface microstructure, and the thickening and twisting of microvessels; the boundary was clearly defined after indigo rouge was sprayed locally. c: Entry into the antrum of the stomach and exit from the mirror showing the mucosal patch of the large curvature of the gastric body and discoloration of the mucous membrane, with a long diameter of about 3.0 cm; surface micro-glandular ducts are sparsely arranged and biopsy traces can be seen in the center (black circle). d: Magnification + NBI suggests that the micro-glandular ducts’ structure disappears upon local exposure, and spiral microvessels can be seen.

**Figure 4:**
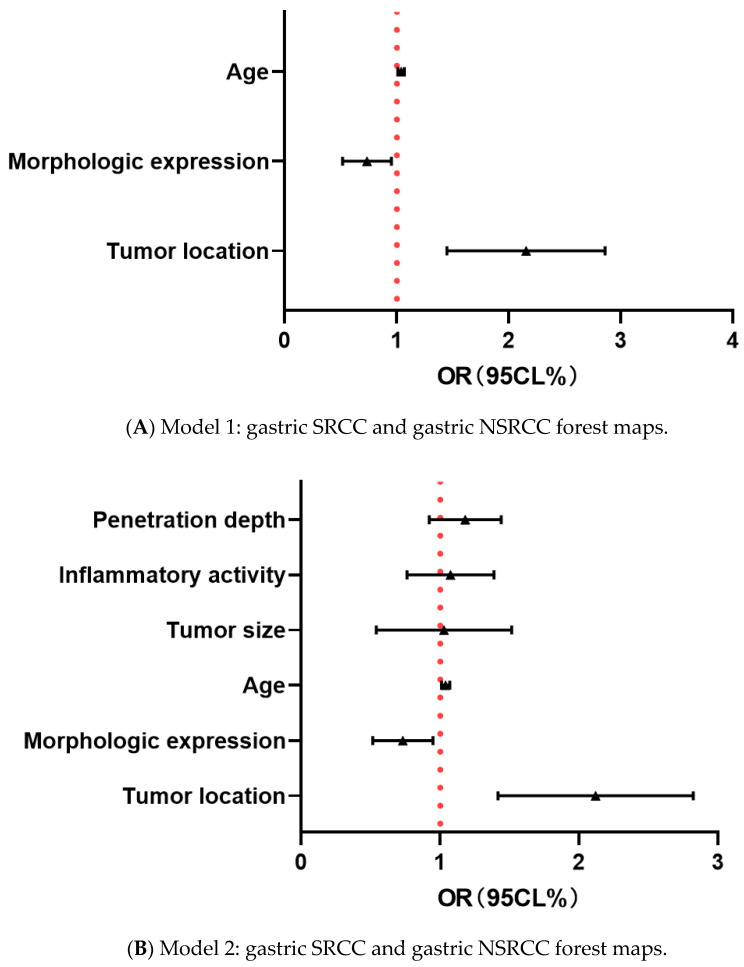

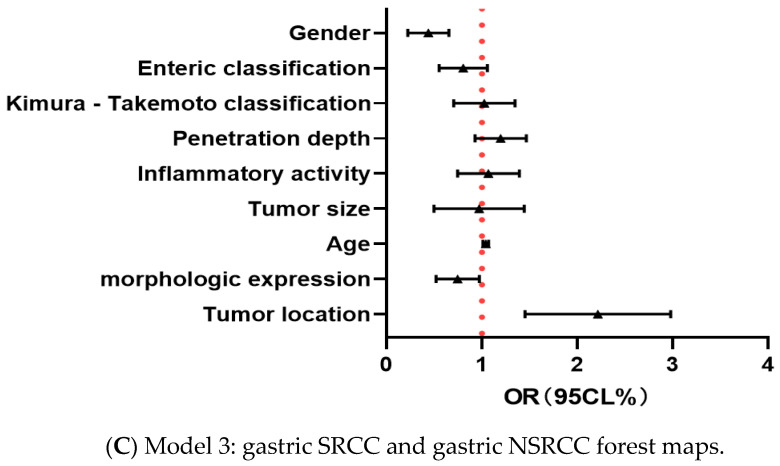
The contents of the forest maps of the gastric SRCC and gastric NSRCC groups include age, tumor site, and morphological manifestations (**A**), plus tumor size, inflammatory activity grade, and depth of invasion (**B**), plus the Kimura–Takemoto classification and enterochemical-grade gender (**C**).

Table 2 After adjusting variables, such as age, morphological manifestation, and tumor site, in logistic regression model 1, it was found that age, morphological manifestation, and tumor site were all correlated with the occurrence of gastric SRCC, and the OR values were 2.076, 0.714, and 1.038, respectively. In model 2, variables such as infiltration depth, inflammatory activity, and tumor size were added. In the end, age, morphological manifestation, and tumor site were still correlated with gastric SRCC, with OR values of 2.041, 0.709, and 1.038, respectively. In model 3, three variables were added,: intestinal grade, atrophy degree, and gender, and the results still show that age, morphological manifestation, and tumor site correlate with gastric SRCC (Figure 4).

Table 3 shows the comparison of the clinical endoscopy and pathology between gastric PSRCC and gastric MSRCC groups. The results show that there are statistical significances for age, the Kimura–Taketo classification, morphological manifestations, and helicobacter pylori infection between the gastric PSRCC and gastric MSRCC groups (*p* < 0.05). The proportion of gastric PSRCC patients under 60 years old was higher than that of gastric MSRCC (76%:48%, *p* = 0.013 < 0.05), and the proportion of gastric PSRCC patients in the C0 stage was higher than that of gastric MSRCC patients (14%:2%, *p* = 0.013 < 0.05). The proportion of flatness and depression in gastric PSRCC patients was higher than that in gastric MSRCC patients (52%:19%, *p* = 0.004 < 0.05), and the proportion of *H. pylori* infection in gastric PSRCC patients was higher than that in gastric MSRCC patients (69%:36%, *p* = 0.001 < 0.05); the difference was statistically significant.

The results of the multifactorial analysis in Table 4 show that H. pylori infection (*p* = 0.104 > 0.05), the Kimura–Takemoto classification (*p* = 0.244 > 0.05), and morphological manifestations (*p* = 0.365 > 0.05) are not independent risk factors for gastric PSRCC (*p* > 0.05); age (OR = 0.233, CL = 0.059–0.930, *p* = 0.039) is the main independent risk factor for gastric PSRCC.

**Figure 5:**
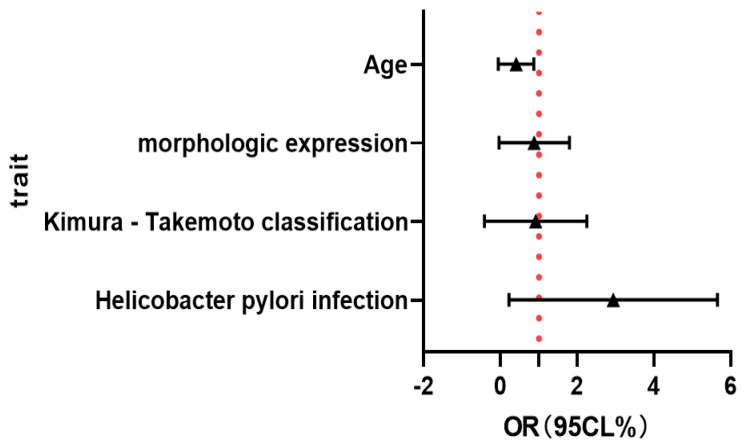
Forest plot analysis of early pure and mixed ring cell carcinomas of the stomach.

**Figure 6:**
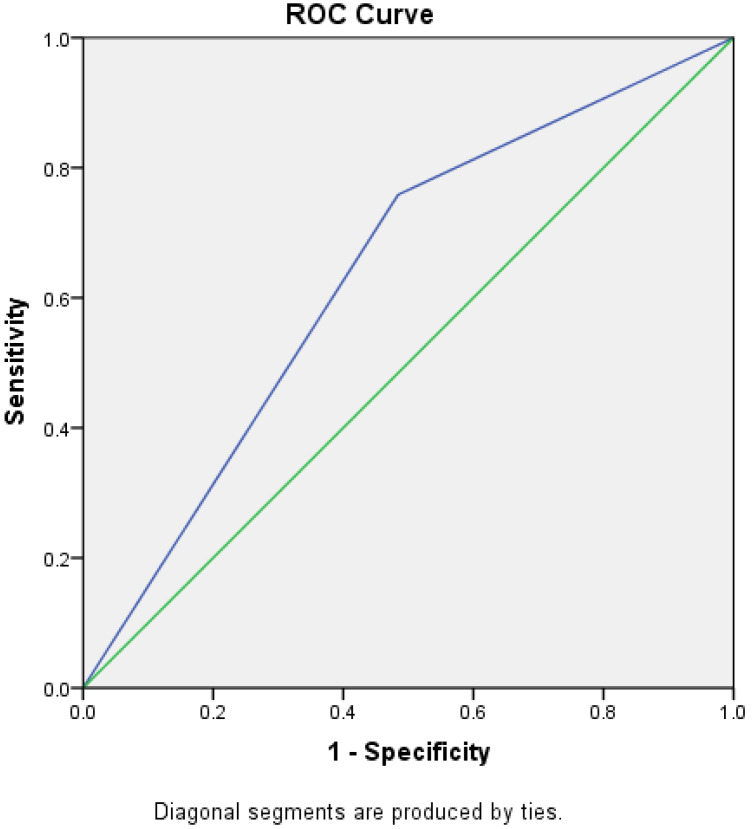
Age ROC curve. The blue line is the efficacy curve for age for the diagnosis of purely indolent cell carcinoma; the green line is the joint model “curve” for age.

A total of 93 SRCC cases were investigated, including 29 gastric PSRCC cases and 64 gastric MSRCC cases. Multiple variables (helicobacter pylori infection, the Kimura–Takemoto classification, morphological manifestations, and age) affecting SRCC in the univariate analysis were further analyzed by multivariate logistic regression analysis. The results show that age (OR = 0.233, CL = 0.059–0.930, *p* = 0.039) is an independent risk factor for gastric PSRCC. FIG 5. The forest map multiple logistic regression equation shows that age is correlated with the EGC differentiation degree. FIG 6. The ROC curve shows that the age AUC is 0.637 (95% CI 0.518–0.756, *p* = 0.035 < 0.05). AUC = 0.637 indicates that acknowledging age is slightly better than guessing to distinguish the degree of differentiation for early gastric PSRCC, but the overall discriminatory power is not strong. 95% CI (0.518–0.756) represents the 95% confidence interval of this AUC value, with the lower limit of 0.518 being greater than 0.5 and *p* = 0.035 < 0.05, suggesting that this AUC value is statistically significant and was not obtained by chance.

The results in Table 5 show that there is no statistically significant relationship between age and tumor site in gastric SRCC patients (*p* > 0.05).

## 4. Discussion

Gastric cancer is highly prevalent in eastern Asia, as represented by China, Japan, and South Korea [11]. Helicobacter pylori infection is an important carcinogenic factor of gastric cancer [12], with the further research on the mechanisms of Helicobacter pylori showing it causes gastric cancer [13]. Gastric cancer’s general incidence has shown a yearly decrease during the past ten years; however, the occurrence rate of gastric signet-ring cell carcinoma has risen, accounting for about 30% of the total number of gastric cancer cases.

Among gastric cancers, there is no significant difference in the clinical presentation between patients with gastric signet-ring cell carcinomas and those with other histological types of gastric cancers. Most patients show no obvious clinical symptoms and signs, and only a few patients present abdominal pain [14], abdominal distension, belching and acid reflux, nausea, vomiting, and other symptoms similar to chronic gastritis or peptic ulcer [15]. On this basis, if the long-term use of anti-inflammatory, antacid, or gastric protection drugs is not an obvious choice, or even when there are symptoms such as a poor appetite, fatigue, upper abdominal pain, or short-term decline in body mass, then gastroscopy is highly likely to be employed for patients with an advanced stage of gastric cancer, including extensive invasion and metastasis.

Endoscopic submucosal dissection (ESD) has become an alternative treatment for patients with early-stage cancer [16]. In general, according to the Japanese cancer treatment guidelines, an undifferentiated histological type is not considered for ESD indications, and recent studies have also reported different outcomes for SRCC and poorly differentiated ESD treatment [17]. Kim et al. found that the poorly differentiated type was significantly associated with submucosal invasion, lymphatic vascular invasion, and the presence of ulcers, implying that the poorly differentiated type is less conducive to ESD treatment than the SRCC histological type, and thus ESD is more suitable for SRCC treatment.

In a retrospective analysis of 75,116 patients with gastric cancer, gastric SRCC patients were younger in age [18] (MD: −4.90, 95% CL: −5.99–3.82, *p* < 0.01) and there were fewer males than gastric NSRCC patients (OR: 0.55, 95% CL: 0.50–0.61, *p* < 0.01), while there were no significant differences in tumor size and lymph node metastasis between gastric SRCC and gastric NSRCC patients. Guo et al.’s study examined 29,851 gastric cancer patients, revealing a notably higher diagnosis rate among younger women carrying the gastric SRCC mutation [19]. There is a research finding that shows that there are more young women in the early gastric SRCC stage [20], while men are the majority in the advanced gastric SRCC stage. The tendency for gastric SRCC to affect young women is widely thought to be linked to sex hormones [21], suggesting a unique pathogenesis distinct from other gastric cancers [22]. In this study, signet-ring cell carcinoma was also found to occur in younger women (*p* < 0.005). As for tumor-prone sites, Xu et al. analyzed the data of 1361 patients with gastric cancer and found that, compared with other types of gastric cancer, gastric SRCC was located ore frequently in the middle and lower-1/3 region of the stomach, and the gastric antrum (including pylorus) was the most common site of SRCC (33.4%) [23], which may be closely related to the higher secretion of gastrin and hormone regulation. This is consistent with the research results in the related article [24]. In this study, we found that the incidence of early gastric-printed signet-ring cell carcinoma was as high as 17% in the upper part of the stomach, 75% in the middle part of the stomach, and 8% in the lower part of the stomach, indicating that early gastric SRCC was more likely to occur in the middle part of the stomach, and the difference was statistically significant. In the study by Zaafouri et al. (2022), SRCC occurred mostly in the lower third of the stomach. However, the present study is a single-center retrospective study, which is subject to some selectivity bias. Therefore, there may be differences in the results. Ostlewait And Yokota stated that the size of gastric NSRCC tumors was less than that of gastric SRCC tumors [25]. However, following our research, there was no notable difference in tumor size between the two groups (*p* > 0.005). The risk of SRCC was 3–5-times higher in patients with systemic gastritis with an atrophic range ≥ 40% than in the normal population [26], and the incidence of SRCC was significantly higher in patients with extensive gastric atrophy (atrophic range > 40%). This paper also shows that the greater the atrophy range in the Kimura–Takemoto classification, the higher the incidence of signet-ring cell carcinomas. Gastric SRCCs rarely presents as simple intramucosal carcinomas (<5%); most of them are T1b (submucosal invasion) or directly progress to T2 (muscular invasion). The depth of invasion (usually T1b) determines the higher risk of metastasis and poor prognosis. In the present study, 34% of the mixed morphological manifestations of gastric PSRCC were higher than 20% of gastric NPSRCC, a statistically significant difference.

At the same time, gastric SRCC was divided into pure SRCC (only SRCC) and mixed SRCC (including other gastric cancer cell types) according to the number of signet-ring cells. Some studies have found that the periodic secretion of estrogen or ER may be a stimulating or inhibitory factor for the occurrence and development of gastric PSRCC. ERβcan inhibits the formation of cell pseudopodia through the mTOR-Arpc1b/EVL signaling pathway, thereby preventing the occurrence and progression of tumors in young gastric PSRCC patients [27]. Estrogen receptor signaling and the mTOR pathway may cross-talk, with ER signaling potentially performing a protective role in pure SRCCs and being synergistic with other pro-oncogenic pathways leading to increased invasiveness in mixed SRCCs. mTOR pathway-aberrant activation in mixed SRCCs is a key prognostic factor, and targeted inhibition may improve the outcomes. Wang et al.’s retrospective study of 1707 patients with early gastric cancer showed that PSRCC was more common in young patients (≤60 years old) compared to MSRCC, as shown in Figure 5 [28]. It is speculated that the expression levels of estrogen or ER in PSRCC and MSRCC patients may also be different, leading to differences in the age of onset and prognosis between the two. This study also found that there were differences in age between PSRCC patients and MSRCC patients. Studies have found that the tumor diameter of gastric MSRCC patients usually exceeds 2 cm and mainly occurs in the lower part of the stomach, and tumor size is one of the important factors affecting the occurrence of pure signet-ring cell carcinomas [29]. However, in this study, these differences were not obvious, as shown in Figure 6. The high incidence of PSRCC in one third of the stomach is the result of the combination of gastric microenvironment disorder (acid deficiency → hypergastrinemia → intestinal metaplasia → carcinogenesis) and local anatomical characteristics (rich blood supply and complex lymphatic drainage). In addition, the prognosis of gastric MSRCC patients is not very optimistic [30] The percentage of indo-ring cells in gastric MSRCC patients is negatively correlated with a poor prognosis [31], which may be due to their invasive nerves and the high number of lymph node metastases. Studies have shown that gastric PSRCC mostly occurs in the inferior 1/3 of the stomach, while gastric MSRCC mostly occurs in the upper 1/3 of the stomach [7]. However, in the present study, pure signet-ring cell carcinomas occurred in 69% and mixed signet-ring cell carcinomas in 78% of the middle of the stomach, *p* = 0.617 > 0.05, which is not statistically significant.

The screening of high-risk SRCC patients should adopt a comprehensive strategy of “endoscopy as the core, molecular detection as the supplement, and preventive intervention throughout the whole process”. In terms of treatment, ESD is suitable for patients with early-stage, low-risk SRCCs (no ulcers, LVI-negative, shallow infiltration, and a negative incisal margin), but the decision needs to be individualized based on endoscopic, pathological, and molecular characteristics. I believe that early gastric SRCC is more suitable for Esd treatment, while progressive gastric SRCC is more suitable for surgical treatment.

The present study still has some limitations: it is a single-center retrospective study that could not avoid selection bias, lacks molecular data, and did not include data such as immunological data; therefore, further studies are still needed to elucidate the relationship between these factors and the pathological subtypes of gastric SRCC. In addition, further studies on the effects of different pathological subtypes of gastric cancer may be needed in the future to guide clinicians in providing individualized medical care for gastric cancer patients.

## 5. Conclusions

Age < 60 years, tumor site in the middle 1/3 of the stomach, mixed type, and female are associated with the development of early gastric SRCC; age < 60 years is associated with the development of gastric PSRCC. Therefore, clinicians need to be aware of these clinical and pathological factors and screen patients with high-risk factors to prevent the development of gastric SRCC.

## Figures and Tables

**Table 1:** Clinical endoscopic and pathological unifactor analysis of gastric SRCC and gastric NSRCC.

Trait		SRCC (n = 93)	NSRCC (n = 331)	OR	*p*
Gender	Male	48 (52%)	233 (70%)	11.456	0.001
	Female	45 (48%)	98 (30%)		
Age	≥60	40 (43%)	252 (76%)	38.394	0.001
	<60	53 (57%)	79 (24%)		
Smoking history	Yes	26 (28%)	103 (31%)	0.343	0.611
	No	67 (72%)	228 (69%)		
Drinking history	Yes	20 (22%)	111 (34%)	0.053	0.461
	No	73 (78%)	220 (66%)		
Family history	Yes	23 (25%)	65 (20%)	1.145	0.311
	No	70 (75%)	266 (80%)		
Kimura–Takemoto classification	CO	5 (5%)	18 (5%)	2.921	0.404
	C1	6 (6%)	14 (4%)		
	C2–C3	40 (43%)	173 (52%)		
	O1–O3	42 (46%)	126 (38%)		
Tumor location	Lower 1/3	16 (17%)	59 (18%)	56.586	0.001
	Middle 1/3	70 (75%)	114 (34%)		
	Upper 1/3	7 (8%)	154 (48%)		
morphologic expression	protrude type	34 (37%)	125 (38%)	9.847	0.007
	Flat and sunken type	27 (29%)	140 (42%)		
	mixed type	32 (34%)	66 (20%)		
Tumor size	≥2 cm	40 (43%)	145 (44%)	0.019	0.906
	<2 cm	53 (57%)	186 (56%)		
Penetration depth	M	53 (57%)	194 (59%)	0.261	0.878
	SM1	26 (28%)	84 (25%)		
	SM2	14 (15%)	53 (16%)		
Helicobacter pylori infection	Free of infection	11 (12%)	32 (10%)	0.386	0.825
	Past infection	39 (42%)	140 (42%)		
	Active infection	43 (46%)	159 (48%)		
Background mucosal atrophy	light	25 (27%)	109 (33%)	0.008	0.931
	medium	31 (33%)	131 (40%)		
	high	37 (40%)	91 (27%)		
	light	20 (22%)	98 (30%)		
Enteric classification	medium	36 (39%)	114 (34%)	0.881	0.348
	high	37 (39%)	119 (36%)		
Inflammatory activity	light	17 (18%)	112 (34%)		
	medium	52 (56%)	76 (23%)	0.492	0.483
	high	24 (26%)	143 (43%)		

Notes: M: intramucosal carcinoma; SM1: carcinoma tissue invades the upper third of the submucosal layer; SM2: carcinoma tissue invades the middle third of the submucosal layer.

**Table 2:** Results of multi-factor analysis of gastric SRCC and gastric NSRCC.

Variable	B	Wald	*p*	Exp.	95% CL
Model 1					
Tumor location	0.730	18.593	0.001	2.076	1.490–2.894
Morphologic expression	−0.338	4.886	0.027	0.714	0.529–0.962
Age	0.037	6.676	0.010	1.038	1.009–1.067
Model 2					
Tumor location	0.713	17.278	0.001	2.041	1.458–2.857
Morphologic expression	−0.343	5.027	0.025	0.709	0.526–0.958
Age	0.037	6.616	0.010	1.038	1.009–1.068
Tumor size	−0.050	0.041	0.840	0.951	0.584–1.549
Inflammatory activity	0.042	0.079	0.779	1.043	0.777–1.400
Penetration depth	0.149	1.768	0.184	1.161	0.932–1.447
Model 3					
Tumor location	0.755	17.899	0.001	2.127	1.499–3.017
Morphologic expression	−0.324	4.268	0.039	0.723	0.531–0.983
Age	0.040	7.700	0.006	1.041	1.012–1.071
Tumor size	−0.111	0.190	0.663	0.895	0.542–1.477
Inflammatory activity	0.035	0.051	0.822	1.036	0.763–1.407
Penetration depth	0.162	1.975	0.160	1.176	0.938–1.475
Kimura–Takemoto classification	−0.009	0.003	0.958	0.992	0.722–1.363
Enteric classification	−0.251	2.394	0.122	0.778	0.566–1.069
Gender	−0.903	12.378	0.001	0.405	0.245–0.670

**Table 3:** Pathological endoscopic unifactor analysis of pure and mixed SRCCs.

Trait		PSRCC (n = 29)	MSRCC (n = 64)	OR	*p*
Gender	Male	16 (55%)	32 (50%)	0.214	0.644
	Female	13 (45%)	32 (50%)		
Age	≥60	7 (24%)	33 (52%)	6.124	0.013
	<60	22 (76%)	31 (48%)		
Smoking history	Yes	6 (21%)	20 (31%)	1.105	0.293
	No	23 (79%)	44 (69%)		
Drinking history	Yes	3 (10%)	17 (27%)	3.109	0.104
	No	26 (90%)	47 (63%)		
Family history	Yes	7 (24%)	16 (25%)	0.008	0.929
	No	22 (76%)	48 (75%)		
Kimura–Takemoto classification	CO	4 (14%)	1 (2%)	10.721	0.013
	C1	3 (10%)	3 (5%)		
	C2–C3	7 (24%)	33 (52%)		
	O1–O3	16 (52%)	26 (41%)		
Tumor location	Lower 1/3	6 (21%)	10 (16%)	0.965	0.617
	Middle 1/3	20 (69%)	50 (78%)		
	Upper 1/3	3 (10%)	4 (6%)		
Morphologic expression	protrude type	6 (21%)	28 (44%)	10.947	0.004
	Flat and sunken type	15 (52%)	12 (19%)		
	mixed type	8 (27%)	24 (27%)		
Tumor size	≥2 cm	20 (69%)	20 (31%)	11.582	0.001
	<2 cm	9 (31%)	44 (69%)		
Penetration depth	M	19 (66%)	34 (53%)	1.378	0.502
	SM1	6 (21%)	20 (31%)		
	SM2	4 (13%)	10 (16%)		
Helicobacter pylori infection	Free of infection	6 (21%)	5 (8%)	17.535	0.001
	Past infection	3 (10%)	36 (56%)		
	Active infection	20 (69%)	23 (36%)		
Background mucosal atrophy	light	8 (28%)	17 (27%)	0.102	0.951
	medium	9 (31%)	22 (34%)		
	high	12 (41%)	25 (39%)		
Enteric classification	light	5 (21%)	15 (22%)		
	medium	10 (31%)	26 (36%)	1.314	0.518
	high	14 (48%)	23 (42%)		
Inflammatory activity	light	5 (38%)	12 (36%)		
	medium	18 (28%)	34 (30%)	0.738	0.691
	high	6 (34%)	18 (34%)		

**Table 4:** Results of multivariate analysis of pure and mixed SRCCs.

Trait	B	Wald	*p*	Exp.	95% CL
Helicobacter pylori infection	1.790	8.442	0.104	5.989	0.791–2.032
Kimura–Takemoto classification (C1)	−1.314	1.358	0.244	0.269	0.029–2.450
Morphologic expression (Bulge type)	−0.578	0.852	0.365	0.561	0.164–1.914
Age (≥60)	−1.455	4.259	0.039	0.233	0.059–0.930

**Table 5:** Relationship between age at onset and tumor site in gastric SRCC patients.

	Lower 1/3	Middle 1/3	Upper 1/3	OR	*p*
Age (≥60)	3	27	10	3.042	0.218
Age (<60)	4	43	6		

## Data Availability

The data that support the findings of this study are available from the corresponding author upon reasonable request.
